# Designing for Effective and Safe Multidisciplinary Primary Care Teamwork: Using the Time of COVID-19 as a Case Study

**DOI:** 10.3390/ijerph18168758

**Published:** 2021-08-19

**Authors:** Lisa Lim, Craig M. Zimring, Jennifer R. DuBose, Jaehoon Lee, Robert J. Stroebel, Marc R. Matthews

**Affiliations:** 1 College of Architecture, Texas Tech University, Lubbock, TX 79409, USA; lisalim8831@gmail.com; 2 College of Design, Georgia Institute of Technology, Atlanta, GA 30332, USA; jennifer.dubose@design.gatech.edu; 3 College of Education, Texas Tech University, Lubbock, TX 79409, USA; jaehoon.lee@ttu.edu; 4 Mayo Clinic, Rochester, MN 55905, USA; stroebel.robert@mayo.edu (R.J.S.); matthews.marc@mayo.edu (M.R.M.)

**Keywords:** healthcare facility design, evidence-based design, architecture, teamwork, patient safety, staff safety

## Abstract

Effective medical teamwork can improve the effectiveness and experience of care for staff and patients, including safety. Healthcare organizations, and especially primary care clinics, have sought to improve medical teamwork through improved layout and design, moving staff into shared multidisciplinary team rooms. While co-locating staff has been shown to increase communication, successful designs balance four teamwork needs: face-to-face communications; situational awareness; heads-down work; perception of teamness. However, precautions for COVID-19 make it more difficult to conduct face-to-face communications. In this paper we describe a model for understanding how layout affects these four teamwork needs and describe how the perception of teamwork by staff changed after COVID-19 precautions were put in place. Observations, interviews and two standard surveys were conducted in two primary care clinics before COVID-19 and again in 2021 after a year of precautions. In general, staff felt more isolated and found it more difficult to conduct brief consults, though these perceptions varied by role. RNs, who spent more time on the phone, found it convenient to work part time-from home, while medical assistants found it more difficult to find providers in the distanced clinics. These cases suggest some important considerations for future clinic designs, including greater physical transparency that also allow for physical separation and more spaces for informal communication that are distanced from workstations.

## 1. Introduction

Effective team-based care improves overall clinical effectiveness across a range of medical settings [1]. It improves patient health outcomes [2,3,4,5,6,7,8], patient safety [9], and population health [10]. Furthermore, teamwork is positively associated with experiences of healthcare professionals, including less emotional exhaustion [11], lower burnout level [12], higher nurse retention [13,14], and increased joy in practice [15]. Fundamentally, team-based primary care facilitates the expertise of a wide range of professionals working in a coordinated way to address both acute and chronic disease.

Recently, healthcare organizations have used the design of the physical setting such as a shared multidisciplinary team room to support better teamwork. Clinic layout and design that brings multidisciplinary staff into physical contact affects the teamwork and communication of healthcare professionals in primary care settings [16,17,18,19,20,21]. Staff workspaces in primary care settings are increasingly designed to foster teamwork by co-locating staff in team rooms where they can more easily communicate and be aware of each other’s activities. 

Ironically, however, recent COVID-19 safety precautions—social distancing, masking, and physical separation—have made these design strategies more difficult to implement, while safety is one of the main goals of teamwork. Due to COVID-19, healthcare administrators made dramatic changes to the built environment of hospitals and clinics to protect against transmission of COVID-19, out of necessity. However, these changes may have unintentional consequences for healthcare teams and teamwork that may well result in negative consequences for other aspects of safety in healthcare as considered in a broader context. 

In this paper, we present a model of how the physical design of space can support teamwork, and we apply the model in two primary care clinics as a case study. We discuss the implications of changes in the process and the built environment to accommodate safe COVID-19 care, the consequences of those changes, and potential mitigation strategies that could be employed during the next pandemic or other disruptions to the healthcare delivery system.

## 2. Design and Teamwork Model

**Figure 1:**
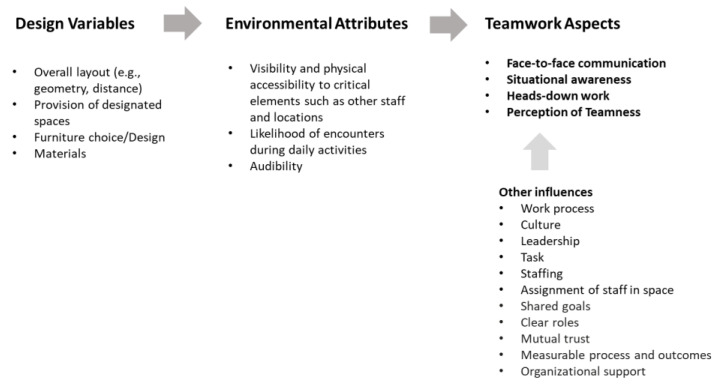
Design and Teamwork Model. The design of space affects the four aspects of teamwork by regulating environmental attributes such as visibility and accessibility. This model can be applied in various settings.

The spatial configuration and design of shared workspaces has been found to influence at least four aspects of teamwork: communication, situational awareness, heads-down work, and perception of teamness [22]. This paper introduces a Design and Teamwork Model that describes relationships between design of space and the four aspects of teamwork (Figure 1). Effective teamwork is certainly influenced by many other factors such as leadership, culture, and trust, but this research only addresses the design of the team spaces; therefore, we limit the discussion to the constructs most impacted by the design.

### 2.1. Communication

Communication is essential for effective teamwork [23,24]. Face-to-face communication, among other types, plays a critical role in most teams, including healthcare settings [25,26,27,28,29,30]. Furthermore, face-to-face communication is significantly associated with the design of the space, including proximity and visibility, while electronic communication, which is more often formal, is not as influenced by physical distance [20].

A body of research has identified design factors that affect face-to-face communication and interactions in various settings. For instance, more frequent communication was reported in spaces with more integrated overall layout [31,32,33], in workspaces with accessible shared team spaces [18,34,35], and in locations that are more accessible to all other spaces [36]. Furthermore, more frequent face-to-face interactions were reported among individuals in close physical distance, such as those who were on the same floor or corridor [37], co-located in the same space [16,38], and proximate to each other’s workstations [19,20]. Additionally, visual connections among individuals, such as visibility between workstations [19,39] or visibility from workstations to overall space [40], were found to be significantly associated with more frequent face-to-face communications.

Face-to-face communication supports rapid information exchange and coordination, especially in healthcare settings where brief encounters (less than five minutes) make up most interactions [41]. The design of shared teamwork spaces, combined with various seating arrangements and workflows, strongly influences the frequency of more impromptu face-to-face communications by creating more opportunities for chance encounters. 

### 2.2. Situational Awareness

Teamwork requires knowledge of how the whole team is functioning, such as seeing when a team member could use help, without their having to ask for help. Situational awareness, “the perception of the elements in the environment within a volume of time and space, the comprehension of their meaning and the projection of their status in the near future” as defined by Endsley [42] (p. 36), is one of critical skills for teamwork [24]. The most important aspects of situational awareness depend on the goals and process of the team. For example, in primary care, the team might need to monitor the activity of the provider to understand when they might be briefed about an upcoming patient, as well as when exam rooms are available.

Built environments affect individuals’ perception of elements (i.e., the first level of situational awareness [42]) by controlling the accessibility of critical elements in the space. A lot of important information about the needs of the team is not directly communicated but arises through observation of the activities going on throughout the space. Previous studies have found that integrated overall layout encouraging movement through the space supports co-awareness of the environment [43,44]; visual access of specific areas (e.g., workstations) improve awareness of patient rooms [45] and other people [46]; and proximity and visual connection to individuals’ targets support awareness of their targets, such as their peers [16,38,45] and patient rooms [20,44,45].

A lot of information is exchanged without requiring explicit communication by passively being able to observe the activities in the space, allowing team members to anticipate needs and proactively volunteer assistance, making things work more smoothly. While digital systems can support situational awareness, it is often simpler and quicker to see critical elements such as other team members and their activities firsthand.

### 2.3. Heads-Down Work

Teamwork also depends on individuals’ ability to do focused work. Collaboration and concentration are related, and both are needed for knowledge work [47]. For instance, while formal and informal communication are critical for coordinating care, medical care requires both routine heads-down work such as entering information into electronic medical records and more complex work such as determining diagnoses and creating treatment plans.

There has been a continued debate among scholars for the last decade about open-plan versus cellular office environments [47]. While the literature assessing the impact of working in open-plan offices is complex and depends on both leadership and self-management practices, it is clear that shared workspaces must accommodate precise, individual work as well as face-to-face communication [48].

Large, open-plan, shared workspace can compromise employees’ ability to concentrate [49], perhaps due to exposure to potential distractions. Workspace design should control visibility and auditory distraction for improved focused work. For instance, higher concentration was reported when individuals saw fewer people in front of their seats [47], as well as at seats which provided individuals with a greater likelihood of seeing their surroundings while not being seen by others [50]. Individual workspaces should allow employees to figuratively block out the sights and sounds of other team members and to signal to others that they are occupied and should not be interrupted. Noise is often mentioned as the most disruptive element, so successful team room design includes separating gathering spaces away from individual desks. Workstations also need to provide employees with access to the tools they need to perform their individual work, such as keeping consistently needed information posted for easy access. 

### 2.4. Perception of Teamness

The design of built environments has representational and symbolic functions, conveying organizational values and images to individuals [51,52,53,54,55]. For instance, the location and views of stairs across floors express organizational dedication to the connections of departments to employees [56], and modern office design changes with an open-office arrangement affect employees’ perception of organizational culture, making it seem less formal and more innovative [57]. 

Likewise, the design of shared workspaces impacts individuals’ perceptions of teamness, defined as having shared goals, clear roles, mutual trust, effective communication, measurable process and outcomes, and organizational support [58]. Individuals who work together at shared spaces (i.e., co-location) reported higher team development measurement scores [21] and considered each other as core team members [17]. More specifically, team spaces where workstations were visually connected were associated with higher teamwork perception scores [59]. In addition, closer meeting areas and a higher proportion of shared service and amenity areas were associated with employees’ having a greater level of perceived support for collaboration [60,61]. The design of team spaces and service areas can place an emphasis on the team over the individual by giving the team ownership of the space. This emphasis sends an important message that the team is an entity and that the team has a shared purpose.

## 3. Case Study: Unpacking the Design and Teamwork Model in Primary Care Settings

The design of workspaces in primary care clinics can facilitate teamwork by creating affordances for these four teamwork aspects (i.e., communication, situational awareness, heads-down work, and perception of teamness), and effectively balancing potentially competing needs such as heads-down work and communication [19,20]. In our previous studies of primary care clinics, we found that the overall co-location of staff predicted improved perception of teamwork [21], proximity among staff workstations facilitated frequent communication [19], and specific design factors such as visibility of exam room doors encouraged situational awareness; teams could easily locate providers and could more easily understand if patients had been seen [20].

### 3.1. Case Study Objectives

In this study, we had the opportunity to continue our research in two clinics, comparing perceptions of teamwork before COVID-19 with teamwork during COVID-19. Both of the clinics studied had teamwork spaces that supported good teamwork through co-location, proximity, visibility and situational awareness prior to the pandemic. In both locations, unavoidable changes were required to the distribution of staff and the work process to keep staff safe, disrupting the very spatial qualities which have been found to support teamwork. The goals of the case study are the following:

1. Examine whether the physical distances and separations among team members resulting from COVID-19 safety precautions affected teamwork experiences of team members;
2. Update and further the Design and Teamwork Model tailored to team-based primary care settings.

### 3.2. Methods

#### 3.2.1. Study Settings 

The two clinics, Clinic A and Clinic B, are Family Medicine clinics of Mayo Clinic, located in Rochester, MN. At Clinic A, comprehensive Family Medicine care is provided for patients from pre-natal to death. This care includes acute care, chronic disease management, wellness, and procedural care. There are four care teams, made up of a total of 18 physicians, 17 nurse practitioners (NPs)/physician assistants (PAs), and 54 nurses, supported by a core team of schedulers, secretaries, clinical pharmacists, and mental health licensed clinical social workers. The patient population is mainly drawn from the city of Rochester and surrounding suburbs, with a large percentage of patients being Mayo employees and their families. 

Clinic B is located on the southern edge of Rochester, serving a patient population drawn from the city of Rochester and a number of smaller, rural communities located to the south of Rochester. Clinic B provides similar comprehensive care for patients as described above. There are two care teams in Clinic B with 8 physicians, 16 NPs/PAs, 28 nurses, and a similar supporting team as Clinic A. 

#### 3.2.2. Team Room Design of the Two Clinics

##### The Use of Team Rooms before COVID-19

Clinic A has four team rooms, each a large space designed to accommodate all the core members of each team (clinicians, registered nurses (RNs)/licensed practical nurses (LPNs)s, and a scheduling specialist). Due to building constraints, each room is slightly different in layout and utilization, but all of these rooms allow for co-location of the team members and place them in close proximity to one another. Each room has evolved gradually over time to best support the team’s personality and workflows. One team room intermingles clinicians and nurses in three distinct pods with low or no barriers between staff. The other three team rooms have staff in different roles, clustered in different areas of the room with standard height cubicle barriers and desks mainly ringing the outside of the room, facing the walls. All rooms have multiple entrance and exit points to allow for team member flow and access to exam rooms and patient waiting areas. 

Clinic B has one large team room. Each of the two care teams occupies one half of the space. The appointment coordinators are in the center of the space and support both teams. The providers in each team are clustered together. The team RNs are also clustered immediately adjacent to the providers. The LPNs are at the periphery of the space, close to the patient hallways, allowing the LPNs to have line of sight and easy access to patient rooms, facilitating their role rooming the patient. Pharmacists, social workers, mental health providers, and dieticians are also located at the periphery of the team room. All team members work in cubicles with chest-high barriers, albeit in proximity of each other. 

##### Changes Resulting from COVID-19 Safety Precautions

Several changes in physical layout, staffing, and clinical processes were enacted in response to the pandemic in an effort to optimize the safety of staff and patients. Non-urgent or elective office visits were deferred. Video or phone visits were encouraged if a face-to-face visit was not absolutely necessary. Staff members not required to perform or facilitate face-to-face office visits were asked to work remotely, including consultative team members such as pharmacists, social workers, and care coordinators, as well as care team nurses who manage phone calls and portal messages. For the remaining onsite staff, workstations were reconfigured to allow a minimum of six feet of distance between individuals, and all staff and patients wore masks. Many providers spent time between patients in private offices rather than team rooms. Electronic communication between team members was encouraged in place of face-to-face conversation. Patients were roomed immediately upon arrival to avoid the use of waiting rooms. As a result of these measures, the co-location and proximity of team members that had previously existed in both clinics was reduced. 

#### 3.2.3. Teamwork Measurements and Analysis 

The teamwork experiences of staff members were measured before COVID-19 and during COVID-19 using two sets of previously validated surveys. The first evaluated survey was the Team Development Survey [62], which consists of 31 items for five constructs (Cronbach’s *a* = 0.97). This study analyzed the three constructs out of five that were investigated in relation to the built environment in a previous study [21]: Communication (14 items), Team Primacy (2 items), and Cohesion (4 items). The two selected clinics had previously administered this survey internally: Clinic A administered the survey in 2016 (Jun.), 2017 (Dec.), and 2020 (Sep.–Nov.), and Clinic B administered it in 2016 (Aug.–Sep.). This study additionally administered the survey in Jan.–Feb. 2021 and compared the results with previously collected datasets, especially comparing pre- and post-COVID-19 (i.e., 2016/2017 vs. 2020/2021).

Another survey instrument measuring teamwork perceptions was adopted from previous studies and included the following constructs: Teamwork Perception [63], Timely Communication [64], Frequent Communication [19], and Awareness [20]. This survey was previously administered only at Clinic B in Nov. 2017 (Cronbach’s *a* for Teamwork Perception = 0.87; *a* for Timely Communication = 0.64; and *a* for Awareness = 0.81). We administered the same survey in Jan.–Feb. 2021 at Clinic B for comparison.

In addition to the two survey instruments, the survey administered in 2021 at the two clinics included open-ended questions: Have there been any changes that were implemented for safety that hinder or support your teamwork? How did that change your daily tasks and patient care? Have precautions for the pandemic affected your ability to do teamwork? If so, in what ways? How might we improve the physical setting or processes to make teamwork easier during the pandemic?

Descriptive statistics were calculated to summarize participants’ demographic characteristics and all outcomes (teamwork subscale scores) at each measurement timepoint (years). For each outcome, general or hierarchical linear modeling (GLM/HLM) examined overall difference between roles (provider, LPN, RN, Other, i.e., role effect), change over time (i.e., time effect), and/or role difference in this change (i.e., role-by-time interaction effect), while properly accounting for the dependency of observations, i.e., individuals (level 1) nested with teams (level 2) if applicable. When an effect was significant at the 0.05 alpha level, marginal means were pairwise compared at a Bonferroni-corrected alpha level. Models also included gender, years of experience in the medical field and at the current site, and/or current team as covariates to produce unbiased effect estimates. All analyses were conducted using SAS 9.4 (SAS Institute, Cary, NC, USA, 2002–2012).

### 3.3. Results of the Case Study

**Figure 2:**
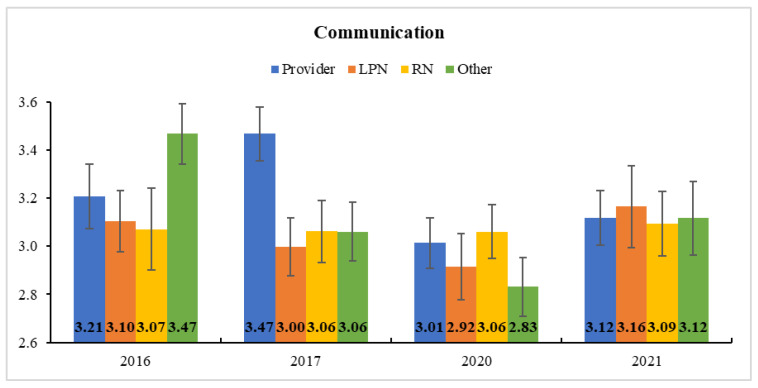
Estimated Marginal Means and Standard Errors of Communication at Clinic A. Providers’ communication perception was significantly lowered in 2020 (after COVID-19) from 2017 (before COVID-19).

The descriptive statistics of survey respondents and team development measures are presented in Table 1. In Clinic A, the subscale scores of team development—*communication*, *team primacy*, and *cohesion—*each decreased after COVID-19 (i.e., from 2016/2017 to 2020), and then bounced back to the previous level in 2021. More interestingly, the role-by-time interaction effect was significant for *communication* in HLM, indicating that it significantly changed during the study period for providers (*F*(3, 239) = 4.30, *p* < 0.01) and “other” roles (*F*(3, 239) = 5.63, *p* < 0.001) but not for LPNs and RNs (both *p* > 0.05). The post-hoc comparison of marginal means further revealed that providers’ *communication* was significantly lowered after COVID-19 from 2017 (*M* ± *SE* = 3.47 ± 0.11) to 2020 (3.01 ± 0.10) (corrected *p* < 0.01; Figure 2). Such a reduction in *communication* by providers was likely a result of the physical isolation of the providers into private offices and work-at-home, and it was recovered in the following year (3.01 ± 0.10) to some degree, showing no significant disparity from the years before COVID-19 (all corrected *p* > 0.05). Neither time nor role-by-time interaction effect was significant for *team primacy* and *cohesion*. The model parameter estimates and Type-3 test results are provided in Table S1.

**Figure 3:**
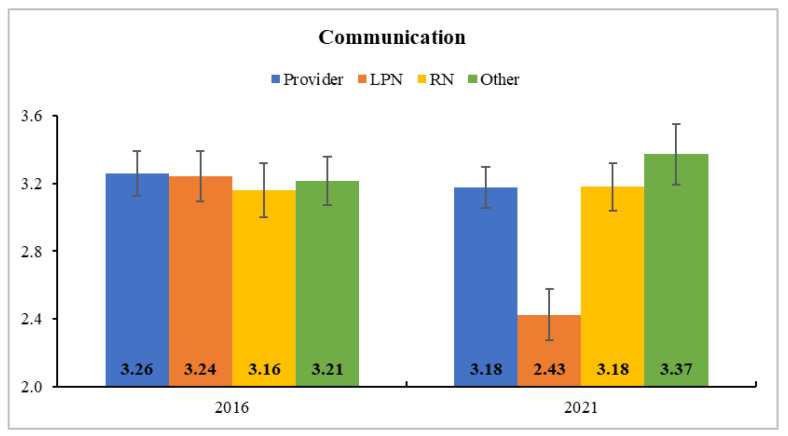
Estimated Marginal Means and Standard Errors of Communication at Clinic B. LPNs’ communication perception significantly dropped from 2016 (before COVID-19) to 2021 (after COVID-19).

**Figure 4:**
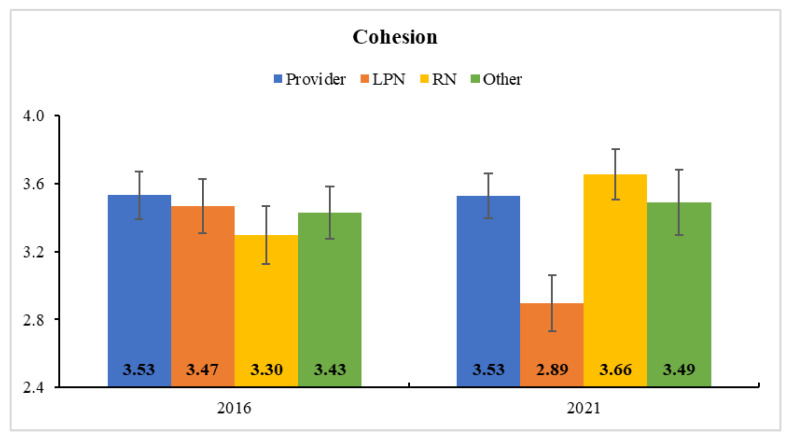
Estimated Marginal Means and Standard Errors of Cohesion at Clinic B. LPN’s self-reported cohesion was also significantly reduced from 2016 (before COVID-19) to 2021 (after COVID-19).

In Clinic B, all subscale scores of team development decreased after COVID-19 (i.e., from 2016 to 2021). The HLM results indicated a significant role-by-time interaction for *communication* and *cohesion* (Table S2). That is, LPNs’ *communication* significantly dropped (*F*(1, 93) = 20.53, *p* < 0.001) from 2016 (3.01 ± 0.10) to 2021 (3.01 ± 0.10; corrected *p* < 0.001), and their *cohesion* was also significantly reduced (*F*(1, 93) = 8.71, *p* < 0.01) from 2016 (3.47 ± 0.16) to 2021 (2.89 ± 0.17; corrected *p* < 0.01). As shown in Figure 3 and Figure 4, however, the scores did not change significantly in other role groups (all *p* > 0.05). 

Clinic B continued to operate as a shared team room, though with staff widely spaced. During the pandemic, some providers and RNs worked from home and patient care and communications shifted to mostly virtual in Clinic B. We suspect that LPNs who usually communicated face-to-face pre-pandemic were affected by the reduction in unplanned encounters. The staff did not encounter each other in their daily activities because their functional paths did not intersect.

**Figure 5:**
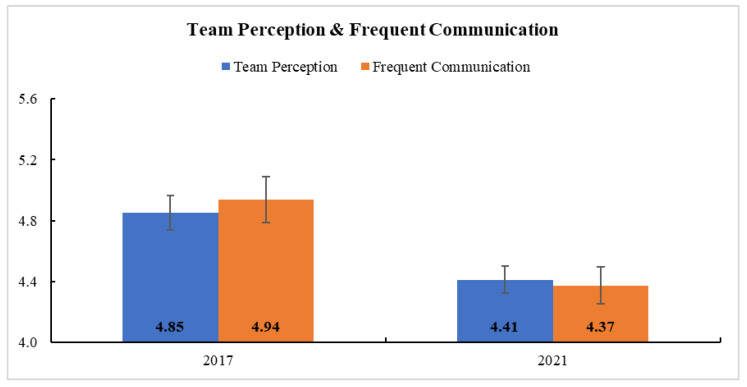
Estimated Marginal Means and Standard Errors of Team Perception and Frequent Communication at Clinic B. Staff members’ self-reported team perception and frequent communication significantly reduced after COVID-19.

Table 2 provides the descriptive statistics of survey respondents and teamwork measures in Clinic B. In general, the teams’ teamwork, as measured by *team perception*, *frequent communication*, *awareness*, and *timely communication*, weakened after COVID-19 (i.e., from 2017 to 2021) (Table S3). Table S3 presents the model parameter estimates and Type-3 test results. The time effect was significant in GLM for *team perception* and *frequent communication*, confirming a significant reduction after COVID-19 in each of those domains (Figure 5). There was no significant interaction between time and role in their teamwork (all *p* > 0.05).

## 4. Discussion

The results of the case study of the two clinics confirmed the role of the built environment in teamwork, especially during the pandemic. Safety precautions such as physical distancing and separation, transition to virtual communication, and removal of visual cues for communication and awareness, negatively affected staff members’ teamwork, including their perception of communication and overall teamwork. 

Using the Design and Teamwork Model, the following section describes how such design changes affected staff members’ teamwork experiences. The case study applies the Design and Teamwork Model in two selected primary care clinics and unpacks the model in detail by revealing a finer level of teamwork needs and identifying some additional aspects of teamwork in relation to design. 

### 4.1. Communication

#### 4.1.1. Face-to-Face Communication Frequency

The survey results illustrate that proximity matters in communication. The increased distance between staff members and their separation into different workspaces was associated with less frequent face-to-face communication. In Clinic B, staffers’ perception regarding the frequency of the communication was statistically lowered in 2021 compared to 2017. More specifically, staff members noted that “there has been less verbal communication because of providers sitting in their offices” (Clinic A, RN), and “I feel that social distancing has reduced communication between members of the teams but I do understand safety is a concern” (Clinic A, LPN). This may be due to the fact that staff members felt that social distance “has made communication more difficult” (Clinic A, LPN). Some staff members noted that “it is difficult to catch them [providers] between patients” (Clinic A, LPN) and “work from a separate room or workstation further from other care team members which required extra steps for communication or extra inbox messages as I wasn’t physically located near the team I needed to communicate with” (Clinic B, Provider). Less verbal communication among staff members might ultimately “delay care and put more work on everyone” (Clinic A, RN), as one staff member noted.

#### 4.1.2. Quick and Timely Communication

Non-co-location and physical distance between staff members seem to hinder the ability to locate staff members to “run something by quickly,” as one RN in Clinic B noted. Working together in the same room with proximity and visibility makes staff members “readily available for questions” (Clinic A, RN), and social distancing “makes it difficult to connect with them face-to-face to have a conversation about patient care that would be more timely than an in-basket” (Clinic A, RN). While electronic messaging can help staff members communicate with each other, “Phone and Skype aren’t effective enough when answers are needed quickly,” as noted by a patient care coordinator in Clinic B. Quick verbal conversations seem to have “saved so much time when we could see the TM [provider of the day] or any provider from our desk and communicate more efficiently” (Clinic B, RN). 

Difficulty locating other staff members appears to affect staff members who need guidance or input from other staff members to proceed with patient care, considering that such comments were made largely by RNs and patient care coordinators. Therefore, making staff members (especially those who are more likely to answer questions and give recommendations) more visible and accessible to other staff members through space design may support staff members to have effective and quick communications with each other.

#### 4.1.3. Informal Communication

One of the major changes adopted in the clinics due to the pandemic was the transition to virtual staff communication regarding patients for both on- and off-site patient care, partly due to the physical distance. Staff members noted that “We are not sitting together as much and the work involves a lot more formal messaging” (Clinic A, Provider), and “More is managed through [electronic] in-baskets back and forth vs. asking a simple question aloud in the team space” (Clinic A, RN). This lack of face-to-face communication made it more difficult for staff members to have collective awareness of patients’ status and context and to prepare before interacting with the patients. The providers often did not have enough time to log into the computer and missed the quick conversations about patients prior to entering the room. They also missed the “blue sheets” briefly summarizing patients’ needs that used to be on the exam room doors.

#### 4.1.4. Group Meetings

The clinics had fewer team meetings during the pandemic, and staff members expressed their concerns regarding this change. For instance, staff members noted that fewer and rushed team meetings “limited our communication” (Clinic A, Provider) and “has hindered discussions and communication that would normally take place” (Clinic B, LPN). One LPN in Clinic B specifically noted that “This has been a challenge and led to some miscommunications on processes and updates.” Furthermore, an RN in Clinic B noted that “The huddles have brought us together in the tough times that we endured.” Therefore, it is strongly recommended to provide a dedicated physical clinic workspace where team huddles can happen (especially during the pandemic, with reasonable distance between staff members).

### 4.2. Situational Awareness

Effective teamwork requires the situational awareness of the staff members so that they are able to respond to each other and to certain events for patient care and coordination. While staff members did not report statistically significant differences between their awareness perceptions pre- and post-pandemic, the survey results revealed the importance of two different types of situational awareness in the clinics: knowing where others are and what they are doing.

#### 4.2.1. Awareness of the Locations of Other Individuals

Working at different sites or rooms made it “harder to find teammates” (Clinic A, Provider). Providers in Clinic A noted that social distancing and working outside of the team room “made it hard to know that they were even present” and “makes it less clear who is actually ‘around’ on the team that day.” This lack of clarity sometimes makes it difficult for staff to locate team members (Clinic A, RN), causing the to “look in a couple of places” (Clinic A, Provider) if they need someone. Therefore, it is recommended to allow staff members who need to work together to share spaces. However, if this is not feasible due to the limited size of spaces, a clear designation of workspaces is needed to improve awareness of the locations of other staff members.

#### 4.2.2. Awareness of the Status of Other Individuals

Another important aspect of situational awareness is knowledge of what other staff members are doing, to help identify whether or not it is a good moment to interrupt and ask a question. For instance, staff members noted that “I frequently do not know if certain team members are working/off” (Clinic A, Provider), and it is challenging to “see if they were behind seeing patients or having a particularly busy day” (Clinic B, Provider).

Interestingly, there was a discrepancy between the perceptions regarding such awareness between providers and other staff members. While providers in Clinic A noted that “I think the team knows providers are sitting in the back of the hallways and know where to find us,” knowing where providers are is not sufficient for other staff members to have a conversation with them. Other staff members commented that “Providers are in their offices so it is sometimes hard to tell if they are on a video/phone visit or just working when their doors are closed” (Clinic A, LPN) and “It is more difficult to tell if the provider has a moment to have a conversation. I tend to spend more time trying to track down a provider to discuss patient care” (Clinic A, RN). Perhaps this discrepancy between providers and other roles is related to their different needs; a person who interrupts and reports to someone needs to know the availability of the person who is interrupted and receives the information.

### 4.3. Heads-Down Work

While staff members expressed their concerns regarding communication and situational awareness with physical distancing and off-site work, some staff members, especially providers and RNs, have noted that their productivity has improved for the heads-down solitary work while working from home or from individual offices. For instance, one provider in Clinic A commented that “I am able to get more done due to fewer distractions now that I am not sitting in a team room (less noise, less interruptions),” and an RN in Clinic A mentioned that “However, I have certainly noticed that I am able to nearly double the amount of work I can do in this setting.” This increased productivity seems to especially be the case for the staff members who need to do both collective and individual work during their clinic days, such as providers and RNs. Even post-pandemic, it is necessary to balance collaboration and heads-down work through the design of clinics. For instance, clinics may provide hot-desking individual work areas separate from team areas, but in close distance and with visibility for awareness and communication, to accommodate focused work tasks.

### 4.4. Teamness

Physical distancing and working from home or individual offices also were associated with lower general team perceptions of staff members. General teamwork perception and team cohesion perception scores of Clinic B were lower in 2021 compared to pre-pandemic years. Staff members commented that “it was much harder to have a sense of a team together” (Clinic B, Specialist) and “it feels like we not working together quite as much” (Clinic A, Provider). 

These changes when working distantly from each other seem to affect staff perceptions regarding relationships with other staff members, as well as their individual feelings. For instance, a provider in Clinic B noted that “moving the MDs has made them seem more ‘separate’ from the rest of us. They had felt more like colleagues and now feel more like bosses/supervisors.” Additionally, an RN in Clinic B shared the feeling of isolation due to physical distancing: “Being spread out creates the feeling of working alone at times. … Now, I often sit at a desk where it’s easy to feel isolated” (Clinic B, RN). 

### 4.5. Identification of Needs and Opportunities for Improved Teamwork through Design

**Figure 6:**
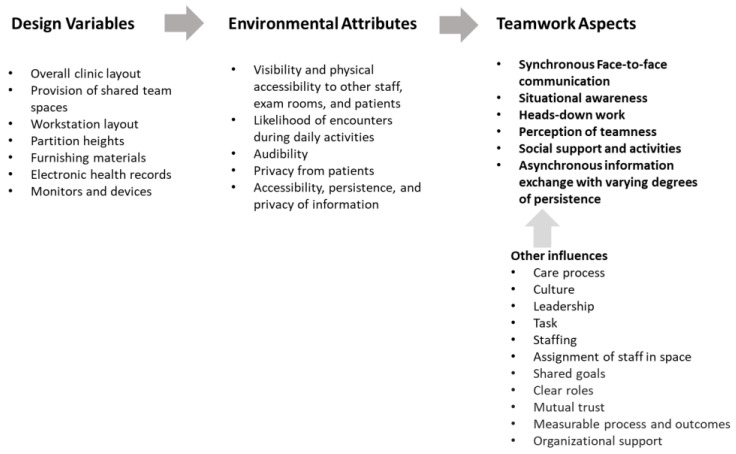
Updated Design and Teamwork Model for Teamwork in Primary Care Settings. The case study furthered and elaborated the Design and Teamwork Model tailored to team-based primary care settings and identified two additional teamwork aspects that can be addressed through design.

While the case study revealed some further insights for the four constructs of the Design and Teamwork Model, especially in primary care settings, it also identified needs and opportunities that can be addressed for improved teamwork through design. The two aspects are design for social support and activities and design for information sharing, both of which can expand the Design and Teamwork Model (Figure 6).

#### 4.5.1. Social Support and Activities

A significant number of staff members pointed out that the social aspect of teamwork is decreasing, due to the pandemic restrictions of separation and limited interactions. Considering that effective teamwork is based on the relationships between staff members, the social aspect of teamwork, enabled through social support and activities, is critical. The design of the built environment seems to affect the social relationships of staff members.

##### Social Support of Team Members

Staff members noted that they “Miss seeing people smile” (Clinic B, Provider) and “Miss the collegiality of working closely with my provider colleagues” (Clinic A, Provider). Furthermore, a lack of social interactions seems to negatively impact their work satisfaction, as noted in one comment: “However, I very much miss the interaction with my teammates and team camaraderie. I would say my joy at work has decreased because of this” (Clinic A, Provider).

##### Work- or Non-Work-Related Social Activities

Pandemic restrictions have also eliminated social activities, including informal communications and gathering for treats or lunches together at or outside of work, as break rooms where people eat and unmask are a significant safety concern during COVID-19. These social activities seem to help develop effective teams, assist workflow, and improve joy at work, as noted by some staff members. Staff members commented that, “These things matter in having a strong team with being able to get together outside of the work area without having to talk about work things” (Clinic B, LPN), and “I miss being able to gather for lunches together and for lunch meetings like roundtables, there are nice times to discuss clinic flow, troubleshoot, informally catch up with one another which also adds joy to work” (Clinic B, Provider). With the pandemic restrictions and social distancing, such social activities were eliminated and they “now eat in our own bubbles away from each other” (Clinic B, Other) and “work becomes work,” making it hard for staff members to “bounce ideas off each other” (Clinic B, RN).

#### 4.5.2. Asynchronous Information Exchange

Another important aspect of teamwork identified in the case study was information exchange for asynchronous communication. 

##### Asynchronous Electronic Communication

The selected clinics made a significant transition to virtual communication and altered their communication modes for safety during the pandemic. Staff members expressed concerns about such transitions. While it is critical for them to obtain information easily when they need it, the electronic communication system made it more difficult to quickly gather and synthesize information in the way that a brief verbal discussion with a team member might. 

##### Accessibility and Persistence of Information

In addition, Clinic B shifted from their previous process of leaving handwritten notes on the door of the exam room to leaving those notes in the exam room to minimize cross-contamination. Staff members report being reluctant to write down sensitive information on the form for other staff members, since patients could see those notes on the forms. As a result, the providers are no longer able to read these notes before they encounter patients. 

With the lack of accessibility and persistence of the information due to the changes, staff members noted that “Since these have been taken away there has been a very large lack in communication and consistency” (Clinic B, LPN). This lack of accessibility and persistence also hindered shared awareness of patients’ status among those who share the workload, leading to inefficiency in work, as noted in the staff comment: “There have been multiple times that 2 nurses were getting immunizations for the same patient” (Clinic B, LPN). 

### 4.6. Summary

Table 3 summarizes the quantitative and qualitative results of the case study, applying the Design and Teamwork Model. The findings of the case study again confirmed the role of spatial attributes on teamwork experiences of the team members. Furthermore, the results of the case study expanded the original Design and Teamwork Model in detail, specifically for primary care settings. It also revealed other aspects of the teamwork that can be supported through design (Figure 6).

## 5. Conclusions

The importance of teamwork is recognized in various settings including aviation, military, and healthcare [13]. Clearly, in each of these settings, safety is of critical importance. The design of the built environment affects various teamwork aspects, as described in our Design and Teamwork Model. For instance, proximity facilitates frequent informal face-to-face communications, visual connection supports awareness of surroundings and specific targets, auditory separation enables heads-down work of individuals, and shared team space symbolically emphasizes teamness.

In this study, we had the opportunity to study two primary care clinics that implemented safety measures in response to COVID-19, which had a significant impact on their built environment, and how they used their spaces. We applied our Design and Teamwork Model to further investigate the relationships between design and space use changes in response to the pandemic and teamwork. 

The results of the case study confirmed that space matters for teamwork. The physical distance and separation among staff members necessitated by COVID-19 precautions decreased the frequency of face-to-face communication and overall perception of teamness. The transition to electronic communication did not support rapid informal communication and could not replace visual cues for communication and awareness. The types of safety measures necessitated by the pandemic adversely impacted teamwork. If not addressed, these design changes might result in a lowered effectiveness of care delivery and decreased overall quality and safety of care. 

Furthermore, the case study provides insights for updating our Design and Teamwork Model. It has proven the applicability of the model in a specific setting and revealed why the four constructs of the model are important and how they are related to space design, especially in primary care settings. It has also identified additional aspects of teamwork that could be improved through design, as illustrated in Figure 6. 

Several design principles for improved teamwork in primary care settings emerge from this and other studies that could be applied to mitigate the impact of the design changes necessitated by the pandemic:

- Provide physical spaces where informal learning discussions can occur among staff members, and provide artifacts and surfaces where those informal notes can be transferred. For instance, designating physical surfaces inside the clinic area with some privacy (especially from patients) for physically sharing and transferring such informal information might help staff members gain a shared understanding of patients.
- Provide physical environments that allow staff members to (visually) check the location and situation of other staff members, even if the spaces are isolated for safety. For instance, transparent partitions or windows between separated spaces might allow for physical distance and separation but maintain a visual connection among staff members, which would lead to an improved awareness of what others are doing.
- Provide a designated space for individual, focused work proximate to the main team spaces. The private and quiet space can allow staff members to be away from other team members and work without interruptions while being closely located to other team members.
- Emphasize teamness by visualizing the team members and their availability through design. Provide shared team rooms where team members can work together, with shared service and amenity spaces near the team rooms.
- Provide a designated area with good cleaning, ventilation, and adequate space for staff members to safely engage in social activities, especially during the pandemic.
- Provide monitors, whiteboards, or bulletin boards that allow for persistent information to be posted about patients and team performance and activities, with attention to privacy and HIPAA.

As discussed, the design and utilization of the built environment is a critical infrastructure that affects teamwork. Design changes can either improve or hinder teamwork. For instance, while the safety precautions in response to COVID-19 are necessary, depending on how the changes are implemented, teamwork could be sacrificed. Our theoretical model of Design and Teamwork could be helpful for evaluating potential consequences. As exemplified in this paper, the Design and Teamwork Model can be applied to teams in various settings, while the priorities of the constructs and needs may vary according to the nature of the teamwork.

## Figures and Tables

**Table 1:** Descriptive Statistics of Survey Respondents and Team Development Measures at Clinics A and B.

	Clinic A: 2016 (*n* = 55)			Clinic A: 2017 (*n* = 72)			Clinic A: 2020 (*n* = 78)			Clinic A: 2021 (*n* = 54)		
Variable	*n*	*M/%*	*SD*	*n*	*M/%*	*SD*	*n*	*M / %*	*SD*	*n*	*M/%*	*SD*
**Team**												
*A*	15	27.3%		20	27.8%		22	28.2%		13	24.1%	
*B*	17	30.9%		19	26.4%		25	32.1%		13	24.1%	
*C*	0	0.0%		18	25.0%		15	19.2%		10	18.5%	
*D*	23	41.8%		15	20.8%		16	20.5%		13	24.1%	
*N/A*	0	0.0%		0	0.0%		0	0.0%		5	9.3%	
**Role**												
*Provider*	14	25.5%	-	22	31.0%		26	33.3%		21	38.9%	
*LPN*	16	29.1%	-	17	23.9%		13	16.7%		8	14.8%	
*RN*	8	14.5%	-	15	21.1%		22	28.2%		14	25.9%	
*Other*	17	30.9%	-	17	23.9%		17	21.8%		11	20.4%	
												
**Communication**	55	3.20	0.48	72	3.17	0.57	78	2.96	0.41	54	3.11	0.32
**Team primacy**	55	2.96	0.69	72	2.97	0.72	78	2.78	0.49	54	3.00	0.40
**Cohesion**	55	3.41	0.52	72	3.39	0.53	78	3.33	0.47	54	3.45	0.46
												
	Clinic B: 2016 (*n* = 48)									Clinic B: 2021 (*n* = 55)		
**Variable**	*n*	*M/%*	*SD*							*n*	*M/%*	*SD*
**Team**												
*CH*	22	45.8%								18	32.7%	
*W*	26	54.2%								20	36.4%	
*N/A*	0	0.0%								17	30.9%	
**Role**												
*Provider*	16	33.3%	-							21	38.2%	
*LPN*	11	22.9%	-							10	18.2%	
*RN*	9	18.8%	-							9	16.4%	
*Other*	12	25.0%	-							15	27.3%	
												
**Communication**	48	3.32	0.39							55	3.10	0.49
**Team primacy**	48	3.29	0.54							55	3.18	0.60
**Cohesion**	48	3.55	0.44							55	3.43	0.50

**Table 2:** Descriptive Statistics of Survey Respondents and Teamwork Measures at Clinic B.

	2017 (*n* = 37)			2021 (*n* = 61)		
Variable	*n*	*M/%*	*SD*	*n*	*M/%*	*SD*
**Role**						
Provider	8	21.6%		21	34.4%	
LPN	9	24.3%		12	19.7%	
RN	8	21.6%		10	16.4%	
Other	12	32.4%		18	29.5%	
**Gender**						
Female	34	91.9%		53	86.9%	
Male	3	8.1%		8	13.1%	
**Experience in the medical field**						
2 years or less	6	16.2%		3	4.9%	
3–5 years	4	10.8%		6	9.8%	
6–10 years	6	16.2%		17	27.9%	
More than 10 years	21	56.8%		35	57.4%	
**Experience at the current site**						
1 years or less	13	35.1%		9	15.0%	
2–3 years	22	59.5%		20	33.3%	
4–5 years	0	0.0%		9	15.0%	
More than 5 years	2	5.4%		22	36.7%	
**Teamwork perception**	37	4.82	0.31	61	4.52	0.54
**Frequent communication**	37	4.86	0.35	61	4.46	0.70
**Awareness**	37	4.34	0.59	61	4.24	0.68
**Timely communication**	37	4.00	0.52	61	3.94	0.67

**Table 3:** Summary of Key Findings According to Design and Teamwork Model.

Teamwork Aspects	Association with Design Attributes
***Communication***	
Face-to-face communication frequency	Communication perception scores (from Team Development Survey) of providers in Clinic A and LPNs in Clinic B significantly lowered post-pandemic. In Clinic B, staffers’ Communication Frequency perception statistically lowered in 2021 from 2017. Staffers stated that physical distance has made communication more difficult, resulting in less verbal face-to-face communication, which might delay care and cause more work for everyone.
Quick and timely communication	Staffers stated that separation and the physical distance between them made it difficult to locate other staffers to quickly run something by them.
Informal communication	Staffers stated that physical distance resulted in more formal electronic communication and less informal face-to-face communication. This made it difficult for staff members to have collective awareness of the patients’ status and context.
Group meetings	A lack of large space for group meetings (with reasonable distance) disabled regular huddles and group meetings. Staffers mentioned the importance of group meetings for teamwork.
***Situational Awareness***	
Awareness of the locations of other individuals	Social distancing and working outside of the team room made it hard to have awareness of other individuals’ presence and locations, making it more effort to find them.
Awareness of the status of other individuals	A lack of visibility due to the physical distance and separation made it hard to know whether others were available for interruption and conversation.
***Heads-Down Work***	
	Staffers, especially providers and RNs, mentioned that separation (working off-site or outside of the team rooms) increased their productivity for the heads-down solitary work.
***Teamness***	
	Team Cohesion (from Team Development Survey) and Teamwork perception scores of Clinic B were statistically lower in 2021 compared to pre-pandemic years. Staffers mentioned that physical distance and separation made them feel isolated and separated from each other.
***Social support and activities***	
Social support of team members	Staffers mentioned that they miss seeing each other, and the lack of social interactions decreased their joy at work. Physical distance and lack of visual connection among staff members made it difficult to have social support as a team.
Social activities	Pandemic restrictions and physical distancing made it hard for staffers to gather for treats, lunches, or lunch meetings. Staffers stated that this made it hard to bounce ideas off each other and decreased joy at work.
***Asynchronous information exchange***	
Asynchronous electronic communication	Staff members mentioned that electronic communication made it more difficult to quickly gather and synthesize information in the way that a brief verbal discussion with a team member might.
Accessibility and persistence of information	Staffers noted that the lack of accessibility and persistence of information created inconsistency and a lack of shared awareness. Communication artifacts, such as vertical surfaces or papers, can affect communication efficiency by regulating the accessibility and persistence of information.

## Data Availability

The data presented in this study are available on request Marc R. Matthews (matthews.marc@mayo.edu).

## Supplementary Materials

The following are available online at https://www.mdpi.com/article/10.3390/ijerph18168758/s1. Table S1: HLM Results on Team Development at Clinic A; Table S2: HLM Results on Team Development at Clinic B; Table S3: GLM Results on Teamwork Measures at Clinic B.
