# Supplementary material for: Designing for Effective and Safe Multidisciplinary Primary Care Teamwork: Using the Time of COVID-19 as a Case Study
Source: Int J Environ Res Public Health. 2021 Aug 19;18(16):8758. doi: 10.3390/ijerph18168758 (PMC8394340; doi:10.3390/ijerph18168758)
Supplement: Supplementary file 1 [file ijerph-18-08758-s001.zip › ijerph-1297130-supplementary.pdf]

Table S1. HLM Results on Team Development at Clinic A: Communication

Effect	Estimate	*SE*	*t*	*p*	*F*	*df* num.	*df* den.	*p*
Intercept	3.11	0.20	15.50	.0000				
**Team**					0.84	4	239	.5010
A	–0.05	0.28	–0.19	.8486				
B	–0.01	0.28	–0.04	.9661				
C	0.11	0.28	0.38	.7047				
D	–0.03	0.27	–0.11	.9096				
N/A (ref.)	0.00							
**Role**					1.49	3	239	.2174
Provider	0.00	0.21	0.01	.9939				
LPN	0.05	0.24	0.20	.8450				
RN	–0.02	0.22	–0.10	.9178				
Other (ref.)	0.00							
**Year**					3.78	3	239	.0112
2016	0.35	0.22	1.63	.1042				
2017	–0.06	0.21	–0.26	.7953				
2020	–0.28	0.21	–1.33	.1854				
2021 (ref.)	0.00							
**Role x Year**					1.98	9	239	.0427
Provider & 2016	–0.26	0.26	–0.99	.3214				
Provider & 2017	0.41	0.25	1.60	.1118				
Provider & 2020	0.18	0.25	0.72	.4710				
Provider & 2021 (ref.)	0.00							
LPN & 2016	–0.41	0.29	–1.41	.1586				
LPN & 2017	–0.11	0.29	–0.39	.6993				
LPN & 2020	0.04	0.29	0.12	.9031				
LPN & 2021 (ref.)	0.00							
RN & 2016	–0.37	0.29	–1.28	.2027				
RN & 2017	0.02	0.27	0.09	.9287				
RN & 2020	0.25	0.26	0.95	.3413				
RN & 2021 (ref.)	0.00							
Other & 2016 (ref.)	0.00							
Other & 2017 (ref.)	0.00							
Other & 2020 (ref.)	0.00							
Other & 2021 (ref.)	0.00							

Table S1. HLM Results on Team Development at Clinic A: Team Primacy (Continued)

Effect	Estimate	*SE*	*t*	*p*	*F*	*df* num.	*df* den.	*p*
Intercept	3.20	0.26	12.20	.0000				
**Team**					1.44	4	239	.2217
A	0.06	0.37	0.16	.8757				
B	0.13	0.36	0.35	.7237				
C	0.19	0.36	0.53	.5970				
D	–0.07	0.36	–0.20	.8436				
N/A (ref.)	0.00							
**Role**					0.95	3	239	.4166
Provider	–0.29	0.27	–1.05	.2925				
LPN	–0.33	0.32	–1.02	.3067				
RN	–0.40	0.29	–1.37	.1724				
Other (ref.)	0.00							
**Year**					2.01	3	239	.1126
2016	0.07	0.28	0.26	.7933				
2017	–0.50	0.28	–1.79	.0746				
2020	–0.48	0.28	–1.71	.0886				
2021 (ref.)	0.00							
**Role x Year**					1.79	9	239	.0707
Provider & 2016	–0.33	0.34	–0.96	.3371				
Provider & 2017	0.61	0.33	1.84	.0675				
Provider & 2020	0.13	0.33	0.41	.6823				
Provider & 2021 (ref.)	0.00							
LPN & 2016	–0.03	0.38	–0.09	.9318				
LPN & 2017	0.53	0.38	1.40	.1625				
LPN & 2020	0.23	0.38	0.61	.5451				
LPN & 2021 (ref.)	0.00							
RN & 2016	–0.11	0.38	–0.28	.7770				
RN & 2017	0.56	0.35	1.58	.1159				
RN & 2020	0.57	0.35	1.64	.1027				
RN & 2021 (ref.)	0.00							
Other & 2016 (ref.)	0.00							
Other & 2017 (ref.)	0.00							
Other & 2020 (ref.)	0.00							
Other & 2021 (ref.)	0.00							

Table S1. HLM Results on Team Development at Clinic A: Cohesion (Continued)

Effect	Estimate	*SE*	*t*	*p*	*F*	*df* num.	*df* den.	*p*
Intercept	3.25	0.22	14.99	.0000				
**Team**					1.25	4	239	.2911
A	0.30	0.30	1.01	.3149				
B	0.42	0.30	1.40	.1630				
C	0.45	0.30	1.50	.1348				
D	0.39	0.30	1.33	.1860				
N/A (ref.)	0.00							
**Role**					3.01	3	239	.0310
Provider	–0.12	0.23	–0.52	.6061				
LPN	–0.33	0.26	–1.26	.2096				
RN	–0.21	0.24	–0.87	.3833				
Other (ref.)	0.00							
**Year**					1.30	3	239	.2742
2016	–0.05	0.23	–0.22	.8266				
2017	–0.41	0.23	–1.79	.0741				
2020	–0.43	0.23	–1.87	.0624				
2021 (ref.)	0.00							
**Role x Year**					1.48	9	239	.1577
Provider & 2016	–0.10	0.28	–0.35	.7303				
Provider & 2017	0.60	0.27	2.19	.0298				
Provider & 2020	0.33	0.27	1.21	.2284				
Provider & 2021 (ref.)	0.00							
LPN & 2016	–0.01	0.31	–0.04	.9692				
LPN & 2017	0.35	0.31	1.11	.2677				
LPN & 2020	0.30	0.32	0.93	.3543				
LPN & 2021 (ref.)	0.00							
RN & 2016	0.15	0.32	0.49	.6256				
RN & 2017	0.27	0.29	0.91	.3620				
RN & 2020	0.43	0.29	1.50	.1358				
RN & 2021 (ref.)	0.00							
Other & 2016 (ref.)	0.00							
Other & 2017 (ref.)	0.00							
Other & 2020 (ref.)	0.00							
Other & 2021 (ref.)	0.00							

Table S2. HLM Results on Team Development at Clinic B: Communication

Effect	Estimate	*SE*	*t*	*p*	*F*	*df* num.	*df* den.	*p*
Intercept	3.45	0.25	13.84	.0000				
**Team**					0.80	2	93	.4537
X	0.04	0.09	0.46	.6459				
Y	–0.27	0.24	–1.13	.2606				
N/A (ref.)	0.00							
**Role**					4.56	3	93	.0050
Provider	–0.19	0.27	–0.73	.4687				
LPN	–0.95	0.28	–3.37	.0011				
RN	–0.19	0.23	–0.85	.3958				
Other (ref.)	0.00							
**Year**					2.48	1	93	.1185
2016	–0.16	0.28	–0.57	.5674				
2021 (ref.)	0.00							
**Role x Year**					4.79	3	93	.0038
Provider & 2016	0.24	0.31	0.77	.4432				
Provider & 2021 (ref.)	0.00							
LPN & 2016	0.97	0.33	2.96	.0039				
LPN & 2021 (ref.)	0.00							
RN & 2016	0.14	0.29	0.48	.6294				
RN & 2021 (ref.)	0.00							
Other & 2016 (ref.)	0.00							
Other & 2021 (ref.)	0.00							

Table S2. HLM Results on Team Development at Clinic B: Team Primacy (Continued)

Effect	Estimate	*SE*	*t*	*p*	*F*	*df* num.	*df* den.	*p*
Intercept	3.56	0.32	11.13	.0000				
**Team**					1.66	2	93	.1962
X	–0.03	0.12	–0.25	.8066				
Y	–0.56	0.31	–1.82	.0722				
N/A (ref.)	0.00							
**Role**					5.95	3	93	.0009
Provider	–0.21	0.34	–0.61	.5413				
LPN	–0.80	0.36	–2.21	.0294				
RN	0.19	0.29	0.66	.5126				
Other (ref.)	0.00							
**Year**					0.28	1	93	.5995
2016	–0.05	0.35	–0.14	.8889				
2021 (ref.)	0.00							
**Role x Year**					1.93	3	93	.1297
Provider & 2016	0.15	0.40	0.38	.7020				
Provider & 2021 (ref.)	0.00							
LPN & 2016	0.26	0.42	0.61	.5463				
LPN & 2021 (ref.)	0.00							
RN & 2016	–0.52	0.37	–1.39	.1677				
RN & 2021 (ref.)	0.00							
Other & 2016 (ref.)	0.00							
Other & 2021 (ref.)	0.00							

Table S2. HLM Results on Team Development at Clinic B: Cohesion (Continued)

Effect	Estimate	*SE*	*t*	*p*	*F*	*df* num.	*df* den.	*p*
Intercept	3.63	0.27	13.52	.0000				
**Team**					1.19	2	93	.3091
X	–0.09	0.10	–0.94	.3521				
Y	–0.34	0.26	–1.30	.1962				
N/A (ref.)	0.00							
**Role**					2.86	3	93	.0411
Provider	0.04	0.29	0.13	.8941				
LPN	–0.60	0.30	–1.97	.0521				
RN	0.17	0.24	0.68	.4951				
Other (ref.)	0.00							
**Year**					0.10	1	93	.7509
2016	–0.06	0.30	–0.20	.8383				
2021 (ref.)	0.00							
**Role x Year**					3.49	3	93	.0188
Provider & 2016	0.07	0.33	0.20	.8442				
Provider & 2021 (ref.)	0.00							
LPN & 2016	0.63	0.36	1.79	.0773				
LPN & 2021 (ref.)	0.00							
RN & 2016	–0.30	0.31	–0.96	.3412				
RN & 2021 (ref.)	0.00							
Other & 2016 (ref.)	0.00							
Other & 2021 (ref.)	0.00							

Table S3. GLM Results on Teamwork Measures at Clinic B: Teamwork Perception

Effect	Estimate	*SE*	*t*	*p*	*F*	*df* num.	*df* den.	*p*
Intercept	4.60	0.17	26.34	.0000				
**Role**					3.98	3	82	.0106
Provider	0.29	0.14	2.03	.0460				
LPN	–0.38	0.17	–2.28	.0254				
RN	0.17	0.17	1.00	.3218				
Other (ref.)	0.00							
**Gender**					0.00	1	82	.9793
Female	0.00	0.15	0.03	.9793				
Male (ref.)	0.00							
**Experience in the medical field**					1.26	3	82	.2946
2 years or less	–0.13	0.16	–0.82	.4170				
3–5 years	–0.26	0.15	–1.70	.0934				
6–10 years	0.03	0.11	0.24	.8095				
More than 10 years (ref.)	0.00							
**Experience at the current site**					1.11	3	82	.3518
1 years or less	–0.20	0.14	–1.41	.1615				
2–3 years	–0.22	0.12	–1.73	.0872				
4–5 years	–0.06	0.17	–0.36	.7228				
More than 5 years (ref.)	0.00							
**Year**					17.29	1	82	.0000
2017	0.55	0.17	3.18	.0021				
2021 (ref.)	0.00							
**Role x Year**					2.00	3	82	.1209
Provider & 2017	–0.47	0.24	–1.93	.0574				
Provider & 2021 (ref.)	0.00							
LPN & 2017	0.15	0.25	0.60	.5520				
LPN & 2021 (ref.)	0.00							
RN & 2017	–0.12	0.26	–0.44	.6586				
RN & 2021 (ref.)	0.00							
Other & 2017 (ref.)	0.00							
Other & 2021 (ref.)	0.00							

Table S3. GLM Results on Teamwork Measures at Clinic B: Frequent Communication (Continued)

Effect	Estimate	*SE*	*t*	*p*	*F*	*df* num.	*df* den.	*p*
Intercept	4.47	0.23	19.14	.0000				
**'Role**					2.12	3	82	.1037
Provider	0.42	0.19	2.18	.0319				
LPN	–0.26	0.23	–1.13	.2605				
RN	0.40	0.23	1.71	.0913				
Other (ref.)	0.00							
**Gender**					0.02	1	82	.8813
Female	–0.03	0.19	–0.15	.8813				
Male (ref.)	0.00							
**Experience in the medical field**					0.65	3	82	.5848
2 years or less	–0.28	0.22	–1.28	.2050				
3–5 years	–0.14	0.21	–0.68	.4986				
6–10 years	–0.01	0.15	–0.07	.9423				
More than 10 years (ref.)	0.00							
**Experience at the current site**					0.77	3	82	.5118
1 years or less	–0.22	0.19	–1.14	.2578				
2–3 years	–0.23	0.17	–1.39	.1672				
4–5 years	–0.02	0.23	–0.09	.9315				
More than 5 years (ref.)	0.00							
**Year**					15.81	1	82	.0001
2017	0.62	0.23	2.70	.0083				
2021 (ref.)	0.00							
**Role x Year**					1.70	3	82	.1731
Provider & 2017	–0.45	0.32	–1.40	.1641				
Provider & 2021 (ref.)	0.00							
LPN & 2017	0.35	0.34	1.03	.3067				
LPN & 2021 (ref.)	0.00							
RN & 2017	–0.14	0.35	–0.39	.6959				
RN & 2021 (ref.)	0.00							
Other & 2017 (ref.)	0.00							
Other & 2021 (ref.)	0.00							

Table S3. GLM Results on Teamwork Measures at Clinic B: Awareness (Continued)

Effect	Estimate	*SE*	*t*	*p*	*F*	*df* num.	*df* den.	*p*
Intercept	4.68	0.26	17.75	.0000				
**Role**					1.20	3	82	.3149
Provider	0.17	0.22	0.80	.4252				
LPN	–0.13	0.25	–0.51	.6090				
RN	0.20	0.26	0.75	.4535				
Other (ref.)	0.00							
**Gender**					2.11	1	82	.1497
Female	–0.32	0.22	–1.45	.1497				
Male (ref.)	0.00							
**Experience in the medical field**					1.86	3	82	.1433
2 years or less	–0.48	0.25	–1.92	.0584				
3–5 years	–0.35	0.23	–1.52	.1336				
6–10 years	–0.21	0.17	–1.26	.2119				
More than 10 years (ref.)	0.00							
**Experience at the current site**					0.49	3	82	.6916
1 years or less	–0.05	0.22	–0.24	.8089				
2–3 years	–0.15	0.19	–0.79	.4307				
4–5 years	–0.27	0.26	–1.06	.2938				
More than 5 years (ref.)	0.00							
**Year**					1.17	1	82	.2832
2017	0.05	0.26	0.20	.8430				
2021 (ref.)	0.00							
**Role x Year**					0.28	3	82	.8409
Provider & 2017	–0.02	0.36	–0.04	.9648				
Provider & 2021 (ref.)	0.00							
LPN & 2017	0.24	0.38	0.63	.5281				
LPN & 2021 (ref.)	0.00							
RN & 2017	0.26	0.39	0.66	.5136				
RN & 2021 (ref.)	0.00							
Other & 2017 (ref.)	0.00							
Other & 2021 (ref.)	0.00							

Table S3. GLM Results on Teamwork Measures at Clinic B: Timely Communication (Continued)

Effect	Estimate	*SE*	*t*	*p*	*F*	*df* num.	*df* den.	*p*
Intercept	4.43	0.25	17.49	.0000				
**Role**					0.34	3	82	.7989
Provider	0.02	0.21	0.07	.9417				
LPN	–0.29	0.24	–1.18	.2420				
RN	0.06	0.25	0.25	.8015				
Other (ref.)	0.00							
**Gender**					1.93	1	82	.1684
Female	–0.29	0.21	–1.39	.1684				
Male (ref.)	0.00							
**Experience in the medical field**					0.96	3	82	.4170
2 years or less	–0.34	0.24	–1.42	.1593				
3–5 years	–0.24	0.22	–1.07	.2870				
6–10 years	–0.13	0.16	–0.81	.4175				
More than 10 years (ref.)	0.00							
**Experience at the current site**					0.88	3	82	.4563
1 years or less	0.01	0.21	0.06	.9558				
2–3 years	–0.20	0.18	–1.11	.2716				
4–5 years	–0.24	0.25	–0.99	.3261				
More than 5 years (ref.)	0.00							
**Year**					0.54	1	82	.4664
2017	0.05	0.25	0.20	.8394				
2021 (ref.)	0.00							
**Role x Year**					0.44	3	82	.7241
Provider & 2017	0.07	0.35	0.21	.8345				
Provider & 2021 (ref.)	0.00							
LPN & 2017	0.31	0.37	0.85	.3967				
LPN & 2021 (ref.)	0.00							
RN & 2017	–0.14	0.38	–0.37	.7120				
RN & 2021 (ref.)	0.00							
Other & 2017 (ref.)	0.00							
Other & 2021 (ref.)	0.00							
